# Ad libitum consumption of protein- or peptide-sucrose solutions stimulates egg formation by prolonging the vitellogenic phase of oogenesis in anautogenous mosquitoes

**DOI:** 10.1186/s13071-022-05252-4

**Published:** 2022-04-12

**Authors:** Ruby E. Harrison, Kangkang Chen, Lilith South, Ange Lorenzi, Mark R. Brown, Michael R. Strand

**Affiliations:** 1 Department of Entomology, The University of Georgia, 120 Cedar Street, 420 Biological Sciences, Athens, GA 30602 USA; 2 Department of Plant Protection, College of Horticulture and Plant Protection, Yangzhou University, Yangzhou, 225009 China

**Keywords:** Mosquito, Oogenesis, Reproduction, Endocrinology, Diet

## Abstract

**Background:**

Anautogenous mosquitoes commonly consume nectars and other solutions containing sugar but are thought to only produce eggs in discrete gonadotrophic cycles after blood-feeding on a vertebrate host. However, some anautogenous species are known to produce eggs if amino acids in the form of protein are added to a sugar solution. Unclear is how different sources of amino acids in sugar solutions affect the processes that regulate egg formation and whether responses vary among species. In this study, we addressed these questions by focusing on *Aedes aegypti* and conducting some comparative assays with *Aedes albopictus, Anopheles gambiae, Anopheles stephensi* and *Culex quinquefasciatus*.

**Methods:**

Adult female mosquitoes were fed sugar solutions containing amino acids, peptides or protein. Markers for activation of a gonadotrophic cycle including yolk deposition into oocytes, oviposition, ovary ecdysteroidogenesis, expression of juvenile hormone and 20-hydroxyecdysone-responsive genes, and adult blood-feeding behavior were then measured.

**Results:**

The five anautogenous species we studied produced eggs when fed two proteins (bovine serum albumin, hemoglobin) or a mixture of peptides (tryptone) in 10% sucrose but deposited only small amounts of yolk into oocytes when fed amino acids in 10% sucrose. Focusing on *Ae. aegypti*, cultures were maintained for multiple generations by feeding adult females protein- or tryptone-sugar meals. Ad libitum access to protein- or tryptone-sugar solutions protracted production of ecdysteroids by the ovaries, vitellogenin by the fat body and protease activity by the midgut albeit at levels that were lower than in blood-fed females. Females also exhibited semi-continual oogenesis and repressed host-seeking behavior.

**Conclusions:**

Several anautogenous mosquitoes produce eggs when provided ad libitum access to protein- or peptide-sugar meals, but several aspects of oogenesis also differ from females that blood-feed.

**Graphical Abstract:**

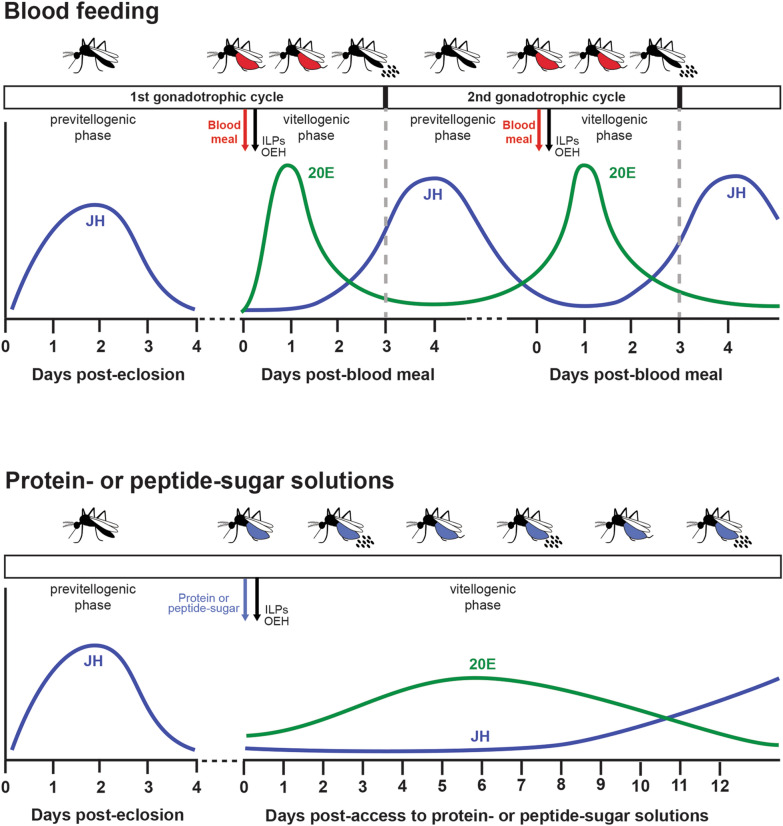

**Supplementary Information:**

The online version contains supplementary material available at 10.1186/s13071-022-05252-4.

## Background

Most mosquitoes (family Culicidae) are anautogenous, meaning that adult females consume blood from a human or other vertebrate host to produce eggs [1]. Anautogenous species usually ingest one blood meal for each clutch of eggs they lay, while sequential blood-feeding underlies how females acquire and transmit blood-borne pathogens between hosts [2]. Mosquitoes in the field additionally consume sugar sources such as nectar that often contains other nutrients including amino acids and proteins [3–6]. Mosquitoes in culture are also usually provided ad libitum access to simplified nectar substitutes such as 5–10% (weight/volume) sucrose or fructose in water [6].

One factor thought to underlie why most mosquitoes are anautogenous is that adult females emerge with insufficient teneral reserves of protein from a detritivorous, aquatic larval stage to activate egg formation [2, 7]. Some species have evolved strategies that increase teneral reserves, which enable females to produce eggs autogenously without blood-feeding [8–15]. However, most mosquitoes overcome this constraint by feeding on blood, which primarily consists of protein by dry weight [16]. Anautogenous species are usually blood-fed in the laboratory on live vertebrate hosts or membrane feeders that are warmed, which serves as a cue for blood-feeding behavior [17]. Protein-rich solutions have been developed as artificial blood meals for use in membrane feeders that, with warming and inclusion of ATP as a phagostimulant, induce *Aedes, Anopheles* and *Culex* spp. to feed and produce eggs with varying levels of efficacy [16–25]. Studies dating back more than a century indicate some anautogenous mosquitoes also consume protein without heating or ATP if added to a sugar solution, which likewise results in egg formation [25–31]. The literature thus overall suggests protein acquisition rather than a specific requirement to blood-feed determines whether female mosquitoes can produce eggs. In contrast, little is known about how the processes that regulate egg formation after blood-feeding are affected by protein or other sources of amino acids in sugar solutions that females feed upon ad libitum like nectar.

Egg formation in response to blood-feeding has been most studied in the anthropophilic vector *Aedes aegypti* where each gonadotrophic cycle consists of two phases [32–34]. The previtellogenic phase begins in the pupal stage when self-renewing germ cells in the ovaries produce cytoblasts. Each cytoblast divides into an oocyte and seven nurse cells that are enveloped by somatic follicle cells to form a primary follicle (= egg chamber) [1, 35–37]. After emerging as an adult, juvenile hormone (JH) released from the corpora allata induces primary follicles to double in size which thereafter enter an indefinite arrest phase unless a female blood-feeds. Blood-feeding stimulates neurosecretory cells in the brain to release insulin-like peptides (ILPs) and ovary ecdysteroidogenic hormone (OEH) that activate the vitellogenic phase by inducing primary follicles to grow and follicle cells to produce ecdysteroids [35–38]. Signaling through the insulin-insulin growth factor, target of rapamycin (TOR), and 20E pathways stimulates the fat body to produce vitellogenin and other yolk components that are packaged into oocytes [32, 34, 39]. 20E titers decline to basal levels by 30 h post blood meal (PBM), terminating yolk protein synthesis, while JH titers rise by 48 h, which enables a second gonadotrophic cycle to occur by stimulating the growth of secondary follicles [32, 34, 39]. Primary follicles become mature eggs that females lay after follicle cells deposit a chorion and degenerate, while secondary follicles become primary follicles that remain arrested unless a female blood-feeds again. ILPs and 20E also stimulate other anautogenous species to produce eggs, which suggests the signaling factors that activate the vitellogenic phase share similarities [39–43].

Here, we examined egg formation in five anautogenous mosquitoes when provided sucrose solutions containing protein, peptides or free amino acids. In *Ae. aegypti*, each solution stimulated yolk deposition into oocytes but the number of eggs females produced differed between individuals that fed once or ad libitum. Egg formation further varied among the five species we examined, while studies that focused on *Ae. aegypti* identified several differences in how ad libitum access to protein- or peptide-sugar solutions affect vitellogenesis compared to a blood meal.

## Methods

### Mosquitoes

The species used in this study were *Ae. aegypti* University of Georgia (UGAL) strain, originally derived from wild-caught adults in Athens, Georgia, and cultured at UGA since the early 1970s [19]; *Ae. albopictus* CDC strain, obtained from the Centers for Disease Control and Prevention (CDC) in Atlanta, Georgia, in 2012; *Culex quinquefasciatus* MR4/BEI strain, obtained by our laboratory from the CDC in 2011; *Anopheles gambiae* G3 strain, obtained from the CDC in 2004; and *An. stephensi* Indian strain, that originated from the Walter Reed Army Institute of Research. All species were housed in an insectary under controlled environmental conditions maintained at 26 °C with ~ 70% relative humidity and a 12 h:12 h light/dark photoperiod.

*Aedes aegypti* larvae were reared in pans at a density of 150/l and fed Cichlid Gold fish food pellets (Hikari). *Ae. albopictus* larvae were reared at a density of 200/l and were provided Cichlid Gold pellets for the first two instars, then maintained until pupation on a rat chow-mix diet consisting of pulverized rat chow pellets (LabDiet 5001), lactalbumin (Sigma) and torula yeast extract (Bio-Serve) in a 1:1:1 by volume ratio. *Culex quinquefasciatus, An. gambiae* and *An. stephensi* larvae were reared at 200/l and fed ground Tetramin fish flakes (Tetra) until pupation. All species were provided ad libitum access to water and 4% fructose-4% sucrose in water provided using cotton wicks for the duration of the adult stage. Eggs for colony maintenance were obtained by providing 3–6-day post-eclosion females defibrinated rabbit blood (HemoStat Laboratories) in membrane feeders (*Ae. aegypti, C. quinquefasciatus, An. gambiae, An. stephensi*) or an anesthetized Sprague-Dawley adult male rat (*Ae. albopictus*). Blood-fed females were provided with a cup lined with wetted paper towel in which to oviposit on days 2–4 post-blood meal. *Aedes* species eggs were dried and maintained in humid conditions for approximately 4 weeks prior to hatching, while *Culex* and *Anopheles* species eggs hatched within 2–3 days following oviposition.

### Protein, peptide and amino acid-sugar solutions

Proteins were solubilized in phosphate-buffered saline (PBS) (NaCl 137 mM, KCl 2.7 mM, Na_2_HPO_4_ 10 mM and KH_2_PO_4_ 1.8 mM) adjusted to pH 7.4 using HCl. Bovine serum albumin (BSA) Fraction V (Research Products International) and rabbit hemoglobin were both solubilized at 200 mg/ml, which mimicked total protein content of whole mammalian blood [44, 45]. Tryptone (Fisher), an enzymatic digest of the milk protein casein, was solubilized at a lower concentration of 50 mg/ml because at higher concentrations mosquitoes were refractory to feeding on the solution. An amino acid stock solution was prepared as previously described in water [46]. To each of these, sucrose 10% w/v was added to produce a protein-, peptide- or amino acid-sugar solution, respectively. FeSO_4_·7H_2_O was added at a final concentration of 100 µg/ml to BSA-, tryptone- or amino acid-sucrose solutions as a source of iron, but was not added to HGB-sucrose as heme already chelates iron.

Mosquitoes were provided protein, tryptone or amino acid-sugar solutions via saturated cotton wick feeders at ambient temperature as either a single meal or ad libitum by replacing feeders daily. For egg formation, gene expression, digestive enzyme activity and ovary ecdysteroidogenesis assays, females were maintained on 4% sucrose-4% fructose from eclosion until 4 days old and then provided protein-sugar solution for the duration of the study. For intergenerational rearing and for host-seeking assays, females were provided protein-sugar meals immediately following eclosion. Mosquitoes were provided one protein-sugar meal wick and one water wick per cage.

*Aedes aegypti* reared intergenerationally on protein-sugar meals were kept in large mesh-sided cages (30 × 30 × 30 cm^3^; BioQuip) at a density of 300–500 individuals per cage. Initial wild-type (F0) adults were sourced from the main *Ae. aegypti* colony at 4 days old and had not previously blood-fed. Adults were given protein-sugar meals ad libitum and allowed to lay eggs for 4 weeks. Eggs were dried and kept in a humid box for 10 days before hatching to produce the ensuing generation. A subset of the first eggs retrieved was used to determine egg viability (hatch rate) for that generation. Larvae were reared at a density of 150 per l of water and fed Cichlid Gold pellets (Hikari) as described above for the main *Ae. aegypti* culture. Adults were provided no other source of nutrients than protein-sugar meals and water.

### Hemoglobin purification

The amino acid content of hemoglobins varies among vertebrates (Dayhoff 1972). Hemoglobins from primates and two-toed ungulates are also known to completely lack the essential amino acid isoleucine [47, 48]. The principal commercially available sources of hemoglobin (human, bovine) are thus isoleucine-deficient with prior studies showing that *Ae. aegypti* females produce no mature eggs when fed either hemoglobin alone [16, 21]. We therefore used hemoglobin from rabbits for this study, which contains all essential amino acids including isoleucine, that we purified from defibrinated rabbit blood (Hemostat Laboratories) [49]. Briefly, rabbit red blood cells (RBCs) were separated from plasma by centrifugation at 2,000 × *g* for 10 min and washed 3 × in PBS to ensure removal of all serum proteins. Oxygen in the tube was replaced with CO_2_, and the contents were gently inverted for 3 min to convert intracellular hemoglobin from its prevalent oxyhemoglobin form (HbO_2_) to the highly stable form carbonylhemoglobin (HbCO), which does not denature at high temperatures. PBS was decanted to isolate packed RBCs, which were lysed by the addition of 1/5 volume dichloromethane (CH_2_Cl_2_, Fisher) and centrifuged at 2000 × *g* for 20 min. The aqueous layer was decanted and heated at 60 °C for 1 h in the dark to denature and precipitate erythrocyte intracellular proteins other than stable HbCO. The final solution was centrifuged at 10,000 × *g* for 20 min to remove precipitates, the supernatant exposed to oxygen and light to reconvert HbCO back to HbO_2_, and the solution lyophilized to obtain pure rabbit hemoglobin powder.

### Follicle development, yolk deposition and egg laying

Bioassays to assess reproductive function included yolk deposition into oocytes, number of maturing follicles, egg lay by females and proportion of viable eggs that hatched into larvae. Maturing ovaries were explanted at different times after blood-feeding, fixed in 4% paraformaldehyde for 5 min, rinsed three times in sterile PBS, stained with DAPI and slide-mounted in 50% PBS-50% glycerol. Yolk deposition into developing oocytes was measured along the anterior-posterior axis of individual oocytes in microns, and three oocytes per ovary pair were measured to generate average yolk deposition for each female [14, 37]. The numbers of primary, secondary and in some cases tertiary follicles were counted while images were taken using a Leica CTRMIC microscope and Leica Application Suite X (LASX) software. To observe oviposition behavior and collect eggs for counting, individual females were placed in 50-ml conical tubes (Falcon). The tube bottom was cut off and topped with nylon mesh pulled taught and glued. Females were given access to protein-sugar meals via a cotton wick inserted vertically into a hole drilled in the cap, while tubes were placed mesh-side down on a wetted paper towel each night to provide an oviposition substrate. Wicks and wetted paper towels were replaced daily until females died.

### Ovary ecdysteroidogenesis and trypsin-like serine protease activity

Ecdysteroid production by ovaries was measured as previously described [50]. Briefly, ovary pairs from two mosquitoes were explanted and incubated in 60 µl Beyenbach saline at 28 °C and 70% RH for 6 h. The resulting supernatant containing secreted ecdysteroids was then collected and frozen at –20 °C until quantification by enzyme-linked immunosorbent assay (EIA) using the primary antibody EAB27, which detects ecdysone and 20E equally and 20E (Sigma) as a standard [50]. Trypsin-like serine protease activity in mosquito midguts was quantified using fresh midgut homogenates from individual females that were incubated with Nα-benzoyl-DL-arginine 4-nitroanilide hydrochloride (BApNA) [51]. Enzyme activity per midgut was measured at 405 nm and quantified using trypsin standards (bovine pancreas, Sigma) using a Biotek Synergy 4 plate reader. Five midgut samples were taken and averaged per treatment and time point indicated.

### Relative and absolute transcript abundance assays

Gene-specific primers were synthesized by IDT (Integrated DNA Technologies) for the following target genes: *Ae. aegypti Hairy* (XM_001662050.2)*, Ae. aegypti Kruppel-homolog 1* (AY433400.1)*, Ae. aegypti ecdysone receptor* (XM_021854525.1), *Ae. aegypti E74* (AF435023.1), *Ae. aegypti E93* (AAEL004572) and *Ae. aegypti vitellogenin A1* (U02548.1) (Additional file 1: Table S1). *Hairy* and *Kruppel-homolog 1* (*Kr-h1*) are transcription factors directly activated by JH binding to its receptor *methoprene-tolerant* (*Met*) [52] and were therefore chosen as targets whose transcriptional upregulation indicates JH signaling. *Ecdysone receptor* (*EcR*) mediates 20E signaling while the ecdysone-induced proteins *E74* and *E93* are expressed downstream of 20E-EcR binding [46]; hence, these three genes were chosen as transcriptional indicators of 20E signaling in *Ae. aegypti*. Finally, *vitellogenin A1* (*VgA1*) upregulation precedes vitellogenin translation and secretion by fat body cells, which as earlier noted is followed by oocyte maturation [53, 54]. Females fed control or protein–sugar meals starting day 4 post-eclosion were sampled on days 1, 2, 3, 4, 5, 7 and 10 during treatment; tissue samples for gene expression consisted of the pooled abdomens of two females with digestive tracts and ovaries removed. Three biological replicates were assessed per treatment and time point. *Aedes aegypti* adult female total RNA was extracted using TRIzol (Ambion) according to the manufacturer’s instructions, and 1 µg RNA per sample was reverse transcribed using a cDNA synthesis kit (Bio-Rad). The relative abundance of JH-responsive (*Hairy* and *Kr-h1*) and 20E-responsive (*EcR*, *E74*, and *E93*) gene expression was assessed by reverse transcriptase quantitative PCR (RT-qPCR) in relation to the reference gene RPL8 [55]. In contrast, *VgA1* expression was quantified by total copy number per sample (absolute expression). *VgA1* copy number was determined by first generating cDNA template from a whole-body female 24 h post-blood meal, which was used to amplify *VgA1* using specific primers followed by cloning into PCR®2.1 TOPO® TA vector (Invitrogen) and transformation into NEB-10β competent *E. coli* (NEB). The resulting plasmid DNA was extracted using a GeneJET Plasmid Miniprep Kit (Thermo) and the *VgA1* insert confirmed by sequencing (Macrogen). Serial dilutions of 10^3^–10^8^ plasmid copies were used to generate a standard curve, which was then used to estimate transcript abundance of *VgA1*. Relative and absolute transcript abundances were assessed using the QuantiFast SYBR Green PCR Kit 4000 (Qiagen), run in quadruplicate technical replicates on a Rotor-Gene Q cycler (Qiagen) under the following conditions: initial denaturation at 95 °C for 10 min, followed by 35 cycles of which denaturation at 95 °C for 10 s, annealing at 55 °C for 15 s and extension at 72 °C for 20 s.

### *EcR* knockdown, methoprene treatment and rapamycin feeding

To knock down *EcR*, double-stranded RNA (dsRNA) was synthesized using a MegaScript RNAi Kit (Ambion) and purified with the MegaClear Transcription Clean-up Kit (Ambion), following the manufacturer’s protocol. cDNA from whole-body female mosquitoes 24 h post-blood meal was used as template for ds*EcR* synthesis, while primers used for dsRNA synthesis are listed Additional file 1: Table S1. As a control, non-specific dsRNA homologous to enhanced green fluorescent protein (*EGFP*) gene was also synthesized using gene specific primers (Additional file 1: Table S1). Newly eclosed female mosquitoes < 24 h old were injected intrathoracically using a Fentojet Express microinjector (Eppendorf) with 0.4–0.6 µl of 2 µg/µl of either ds*RNA* (ds*ECR* or ds*EGFP*). Following injection, 15–20 female mosquitoes were placed into small cages (5 × 5 × 8 cm^3^) with an equivalent or greater number of males to ensure mating. Females were provisioned with one cotton wick saturated with water and one wick saturated with BSA-sucrose solution, changed daily. At 5 days post-injection, females were cold-anesthetized and ovaries explanted for yolk deposition measurement while pelts (abdomens without GI tract or ovaries) from the same females were used to measure *EcR* knockdown. Pelt total RNA was extracted using a Quick-RNA Mini-prep Kit (Zymo) and RT-qPCR run using the same conditions and *EcR* primers described previously.

Methoprene (Zoecon) was solubilized in absolute ethanol to a concentration of 1 µg per µl. A volume of 0.20–0.25 µl was then applied to the abdomen of cold anesthetized females resulting in a dose of 200–250 ng while controls were treated with an equivalent volume absolute ethanol. Females were then placed in small cages (5× 5 × 8 cm) and provided separate cotton wicks saturated with water or BSA-sucrose. Total RNA was extracted 24 h post-treatment from a subset of females and RT-qPCR assays was run to determine relative expression levels for *Hairy* using the same methods described in the previous section. Other females were sampled 5 days post-treatment and yolk deposition into oocytes was measured.

A 40 mM rapamycin (LC laboratories) stock solution in dimethylsulfoxide (DMSO) was diluted to a final concentration of 400 µM in 10% sucrose made fresh daily and fed to newly eclosed *Ae. aegypti* for 2 days. Adults were then provided a BSA-sucrose solution containing 400 µM rapamycin, again made fresh daily from the stock solution, for 5 days and yolk deposition into oocytes measured. Controls were provided 10% sucrose and BSA-sucrose solutions each containing 1% DMSO since this was the concentration of solvent present in treatment solutions. Yolk deposition was assessed for females sampled 5 days following the introduction of BSA-sucrose, i.e. at 7 days total of rapamycin treatment.

### Host-seeking assay

Attraction of *Ae. aegypti* females to a human was assessed using a recently described behavioral “host proximity” assay [56]. Briefly, newly eclosed *Ae. aegypti* females were housed in mesh-topped cardboard cups (10 × 13 × 13 cm^3^) at a density of ten females per cage. Five replicate cages, i.e. a total of 50 females, were set up for each treatment. Once a day at 4 p.m. a member of our team hovered their hand closely over each cage for 1 min and tallied the total number of females that flew to the top of the cage and began probing. Host attraction was recorded for 21 days or until cages contained fewer than three surviving females.

### Data analysis

Proportional data were analyzed by Chi-squared test. The days required by females to oviposit and egg clutch sizes were assessed for individually housed females, individuals serving as the unit of replication, with 30 females total per treatment. Oviposition time and egg clutch size were compared between G0 (wild-type) and tryptone F6 females using a Student’s t-test for each meal (live rat, rabbit blood), following initial confirmation of normality and homogeneity of variances of data via Shapiro-Wilk and Bartlett’s tests, respectively. Student’s t-tests were used to analyze dsEcR, methoprene, and rapamycin treatment data by comparing gene copy number and yolk deposition to the negative control. Survival curves were analyzed using a log-rank (Mantel-Cox) test. The proportion of females that was attracted to human was analyzed using a repeated measures linear mixed effects regression. Here treatment (meal) was designated as a fixed effect, and its interaction with time (days elapsed) assessed. Replicates consisted of cohorts of ten mosquitoes, with five replicate cohorts per treatment; hence, each cohort was treated as the random effect in the model. Analyses presented compare the linear regression slopes among treatments.

Data analyses were performed using R v4.2.1 and GraphPad Prism v9.0.1. Graphs were generated using GraphPad Prism, while text resizing and alignment of graphs were done using Adobe Illustrator v24.0.1. Images of mosquito digestive tracts were taken using a Leica MZ FLIII stereo microscope and Leica Application Suite X (LASX) software while images of mosquito ovaries were captured using a Leica DMRE epiflourescent microscope. Resulting images were exported to Adobe Photoshop v23.0.1 for cropping followed by figure assembly in Adobe Illustrator.

## Results

### Protein-sucrose solutions stimulate anautogenous mosquitoes to produce eggs

**Fig. 1:**
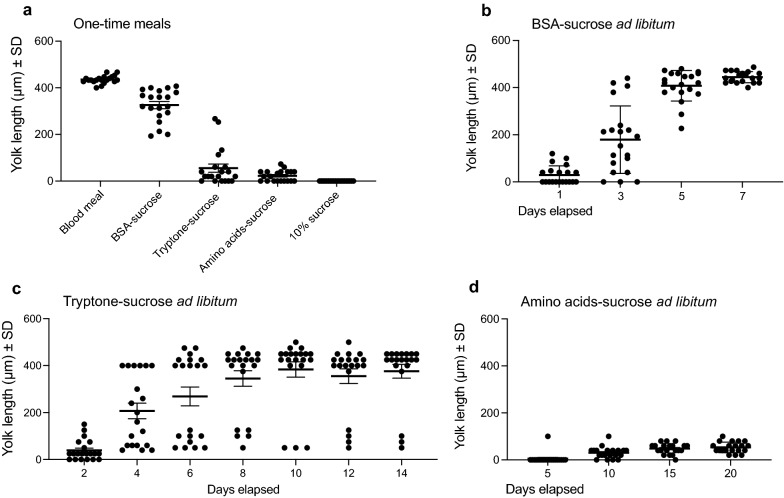
BSA-, tryptone- and amino acid-sucrose solutions variably promote yolk deposition in the oocytes of *Ae. aegypti* females*.* (**a**) Yolk deposition 48 h after feeding once to repletion on rabbit blood, BSA-, tryptone- or amino acid-sucrose. Yolk deposition was assessed by measuring yolk length in oocytes along the anterior–posterior axis. Dots indicate average yolk length in oocytes of one female; group mean and standard error are indicated for each time point. **b**–**d** Oocyte maturation at different times when *Ae. aegypti* females were provided ad libitum access to BSA- (**b**), tryptone- (**c**) or amino acid- (**d**) sucrose. Yolk length was measured every 2 or 5 days by dissecting females as described in (**a**). Dots indicate average yolk length for one female; group mean and standard error are indicated for each time point

Several early studies reported examples of anautogenous mosquitoes laying eggs after consuming protein in sugar solutions [26–29]. More recently, field-collected *An. darlingi* and laboratory-reared *Ae. albopictus* were also found to lay eggs after consuming a single meal of 5–10% sucrose and 10–40% bovine serum albumin (BSA) solution [30, 31]. We began this study by allowing starved *Ae. aegypti* to feed to repletion on a single meal, which was either rabbit blood delivered using a heated membrane feeder (positive control) versus three protein sources solubilized in PBS with 10% sucrose: BSA, tryptone (peptides) or free amino acids. While offered for the same period of time as blood (1 h), protein-, peptide- and amino acid-sucrose solutions were provided at ambient temperature via saturated cotton wicks without the phagostimulant ATP or heating. Most females fed to repletion for each treatment. Dissecting females at 48 h post-feeding showed that most oocytes in the ovaries from blood-fed females were fully mature (400–600 µm yolk length) while females that consumed a BSA-sucrose meal contained eggs that were nearly mature (200–400 µm) (Fig. 1a). In contrast, females that consumed a tryptone-sucrose or amino acid-sucrose meal contained oocytes with little (< 100 µm) or no yolk (Fig. 1a).

We next provided large cohorts (~ 200) of *Ae. aegypti* females in cages ad libitum access to each protein-, peptide- or amino acid-sucrose solution from wicks over several days, which mimicked how sugar solutions are normally provided to mosquitoes in culture or how nectar sources are often available to mosquitoes in the field. In these assays, each cohort of females was also provided ad libitum access to a water wick. Dissecting females at regular intervals and inspecting their ovaries showed that all individuals provided BSA-sucrose and water contained mature eggs after 7 days (Fig. 1b). Most individuals provided tryptone-sucrose and water also contained mature eggs (≥ 400 µm) after 8 days, whereas most individuals provided amino acids-sucrose and water contained primary follicles in which only small amounts of yolk (< 100 µm) were present in oocytes after 10 days (Fig. 1c, d).

**Fig. 2:**
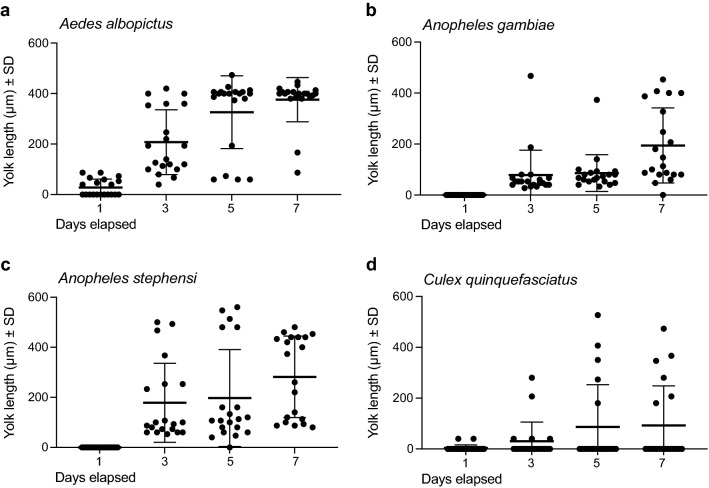
BSA-sucrose variably promotes yolk deposition into oocytes from *Ae. albopictus* (**a**), *An gambiae* (**b**), *An. stephensi* (**c**) and *C. quinquefasciatus* (**d**). Four-day-old females were provided ad libitum access to BSA-sucrose for 7 days. Twenty females per time point were destructively sampled and yolk deposition measured as described in Fig. 1

**Fig. 3:**
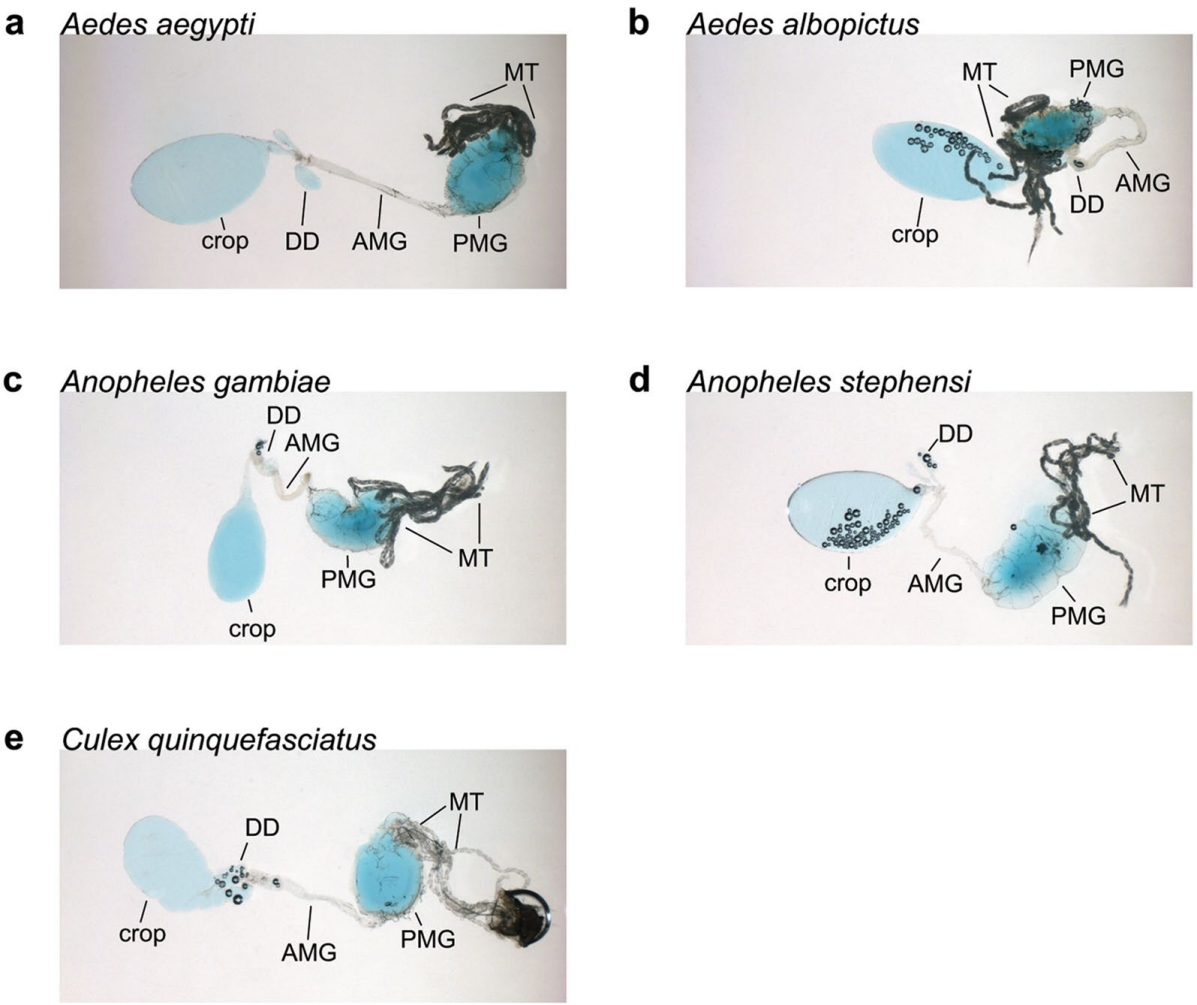
*Aedes aegypti* (**a**)*, Ae. albopictus* (**b**)*, An. gambiae* (**c**)*, An. stephensi* (**d**) and *C. quinquefasciatus* (**e**) shunt BSA-sucrose solutions into both the crop and midgut. Each image shows an explanted digestive tract from a female provided ad libitum access to BSA-sucrose containing blue food dye for 24 h. The crop, dorsal diverticula (DD), anterior midgut (AMG), posterior midgut (PMG) and Malpighian tubules (MT) are indicated; the hindgut is visually obstructed by the MT but contained only trace amounts of blue BSA-sucrose, similar to the foregut

We also provided four other anautogenous species (*Ae. albopictus, An. stephensi, An. gambiae, C. quinquefasciatus*) ad libitum access to BSA-sucrose and water, because this treatment elicited the strongest egg formation response in *Ae. aegypti*. Most *Ae. albopictus* females produced mature eggs after 7 days (Fig. 2a). In contrast, only 50% of *An. stephensi*, 25% of *An. gambiae* and 15% of *C. quinquefasciatus* did so (Fig. 2b-d). While anautogenous mosquitoes direct sugar meals to the crop, females shunt blood or protein-rich artificial blood meals to the midgut [57]. Each of the species we examined took BSA-sucrose into both the crop and midgut simultaneously (Fig. 3). Daily dissections further indicated that females always had full crops and midguts, which strongly suggested: (i) feeding on this protein-sucrose solution occurred daily, if not more frequently, and (ii) differential feeding was not responsible for the two *Anopheles* spp. and *C. quinquefasciatus* maturing fewer eggs than *Ae. aegypti* and *Ae. albopictus*.

Finally, we compared hatch rates of eggs laid by females maintained in cages. Control cages were provided 10% sucrose and water ad libitum for 4 days post-emergence followed by blood-feeding while treatment cages were provided ad libitum access to BSA- or tryptone-sucrose and water. We also created a third treatment group that was provided ad libitum access to hemoglobin (HGB)-sucrose plus water because HGB is the most abundant protein in vertebrate blood by dry weight (80%) [58] and therefore represents a major portion of the diet anautogenous adult females consume. Only the two *Aedes* species laid viable eggs when fed tryptone-sucrose (Table 1). All species except *C. quinquefasciatus* laid viable eggs when fed BSA-sucrose, while all but *An. gambiae* and *C. quinquefasciatus* laid viable eggs when fed HGB-sucrose (Table 1). The proportion of eggs that hatched also varied among treatments with tryptone- and HGB-sucrose yielding higher hatch rates than BSA-sucrose but lower hatch rates than blood-feeding (Table 1).

**Table 1:** Proportion of laid eggs that hatch when mosquitoes in large cages consumed a single blood meal or were provided BSA-, hemoglobin- or tryptone-sucrose solutions ad libitum

Proportion hatched (total eggs laid)^1^				
*Aedes aegypti*				
Blood meal (rabbit)	BSA-sucrose	Hemoglobin-sucrose	Tryptone-sucrose	
0.72 (3462)	0.11 (1493)	0.59 (511)	0.32 (480)	*p* < 0.0001
*Aedes albopictus*				
Blood meal (rat)	BSA-sucrose	Hemoglobin-sucrose	Tryptone-sucrose	
0.81 (385)	0.23 (2612)	0.60 (400)	0.25 (1042)	*p* < 0.0001
*Anopheles gambiae*				
Blood meal (rabbit)	BSA-sucrose	Hemoglobin-sucrose	Tryptone-sucrose	
0.74 (833)	0.51 (745)	NA	NA	*p* < 0.0001
*Anopheles stephensi*				
Blood meal (rabbit)	BSA-sucrose	Hemoglobin-sucrose	Tryptone-sucrose	
0.86 (1517)	0.38 (761)	0.62 (679)	NA	*p* < 0.0001
*Culex quinquefasciatus*				
Blood meal (rabbit)	BSA-sucrose	Hemoglobin-sucrose	Tryptone-sucrose	
0.68 (1274)	0.00 (116)	NA	NA	*p* < 0.0001

^1^NA indicates no eggs were laid by females. Statistical significance is indicated to the right (${\rm X}^{2}$ test)

### *Aedes aegypti* can be maintained for multiple generations by feeding females protein-sucrose solutions

We assessed whether *Ae. aegypti* can be maintained in large cages without blood-feeding if provided ad libitum access to a BSA-, HGB- or tryptone-sucrose and a separate water source. For the first generation, eggs laid by females from our main culture were hatched and reared on a rat chow-based diet used to maintain our principal colony. We placed ~ 200 of the emerging adults in cages provisioned with each protein-sucrose solution, water and oviposition containers. Laid eggs were then hatched to produce the next generation of adults. A population fed tryptone-sucrose was maintained for ten generations while populations fed BSA- or HGB-sucrose were maintained for five generations. Notably, hatch rates increased for each treatment, which resulted in higher proportions of eggs hatching in the last generation than the first (Table 2). Hatch rates for the fifth generation fed HGB-sucrose and tenth generation fed tryptone-sucrose also did not differ from the hatch rate for eggs laid by blood-fed females, but hatch rates for the fifth generation fed BSA-sucrose remained lower (Table 2). Replacing oviposition substrates daily indicated that females laid eggs ~ 6–20 days post-emergence, but in group cages we could not determine whether individual females laid eggs once or multiple times. We also did not measure any other traits besides hatch rates but no obvious intergenerational differences in adult survival, size, feeding behavior or egg laying were observed relative to our conventional culture where females were blood-fed.

**Table 2:** Progeny hatch rates for *Ae. aegypti* intergenerationally reared on protein–sugar meals

Generation	BSA-sucrose	Hemoglobin-sucrose	Tryptone-sucrose
F0	0.09 (768)	0.56 (575)	0.53 (225)
F1	0.15 (486)	0.55 (503)	0.58 (273)
F2	0.22 (394)	0.57 (495)	0.62 (302)
F3	0.26 (562)	0.72 (392)	0.70 (261)
F4	0.22 (627)	0.72 (337)	0.65 (355)
F5	0.44 (719)	0.86 (414)	0.39* (128)
F6	NA	NA	0.63 (192)
F7	NA	NA	0.50 (208)
F8	NA	NA	0.66 (257)
F9	NA	NA	0.72 (248)
F10	NA	NA	0.67 (319)

Proportion of hatching eggs is indicated followed by the total number of eggs tested in parentheses. The proportion of eggs that hatched significantly differed between F0 and F5 for BSA- (*P* < 0.0001) and hemoglobin-sucrose (*P* < 0.0001) as well as between F0 and F10 for tryptone-sucrose (*p* = 0.0008) (${\rm X}^{2}$ test)

*Second egg clutch hatched rather than the first, likely causing unusually low hatch rate

**Fig. 4:**
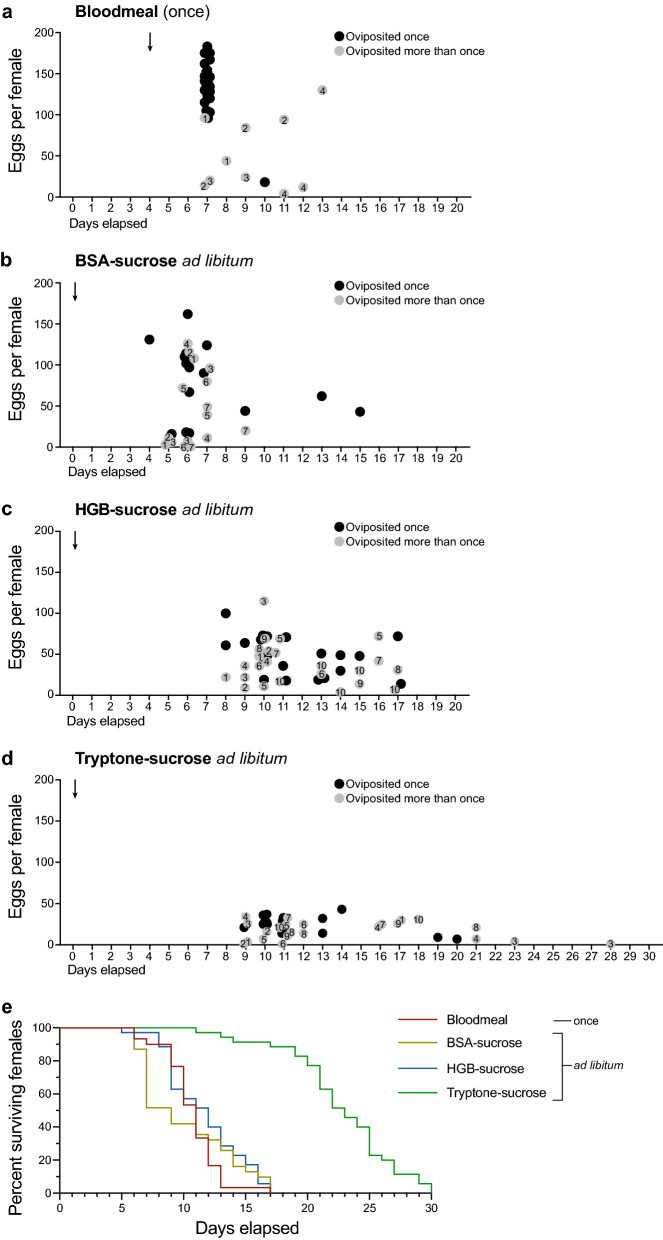
Egg laying differs among *Ae. aegypti* females that blood-fed once (**a**) or had ad libitum access to BSA- (**b**), HGB- (**c**) or tryptone-sucrose (**d**). Newly eclosed females were individually housed in small cages with oviposition substrates replaced daily until death. Females were blood-fed 4 days post-eclosion while protein-sucrose solutions were provided on day 1 post-emergence. Circles indicate the number of eggs laid by an individual female: black circles indicate the female laid eggs only once while gray circles indicate females that laid eggs more than once. Numbers inside each gray circle indicate the female while the y axis indicates the number of eggs laid on a given day. (**e**) Kaplan-Meier plot showing the survival of the same females used to assess egg laying in a–d. Total number of females monitored for each treatment was 30 (blood), 32 (BSA-sucrose), 35 (HGB-sucrose) and 35 (tryptone-sucrose)

To assess when females laid eggs, pupae from the general culture were allowed to emerge and mate in cages provisioned with BSA-, HGB- or tryptone-sucrose and water for 3 days, followed by separating adult females into individual cages that were also provisioned with each sucrose solution, water and an oviposition cup until death. Controls were allowed to emerge and mate in cages provisioned with 10% sucrose and water, blood-fed 4 days post-emergence and then separated into individual cages provisioned with 10% sucrose, water and an oviposition cup. As expected, most blood-fed control females laid a single clutch of eggs 7 days post-emergence (3 days PBM), although four individuals laid two or three smaller clutches 3–9 days PBM (Fig. 4a). Most females fed BSA-sucrose laid eggs 4–9 days post-emergence with most also ovipositing once (Fig. 4b). In contrast, most females fed HGB- or tryptone-sucrose laid eggs 9–17 days post-emergence with some laying eggs only once and others laying eggs 2–4 times (Fig. 4c, d). The number of eggs laid within a 24-h period (average clutch size) was highest for blood-fed females (109.9 eggs) followed by BSA-sucrose (64.2 eggs), HGB-sucrose (43.1 eggs) and tryptone-sucrose (20.6 eggs). The average total number of eggs laid per female over their lifetime was also highest for control females (133.5 eggs) followed by BSA-sucrose (92.9 eggs), HGB-sucrose (63.2 eggs) and tryptone-sucrose (28.4 eggs). Reciprocally, females maintained on tryptone-sucrose lived longer than females fed HGB-sucrose, BSA-sucrose or blood (Fig. 4e).

**Fig. 5:**
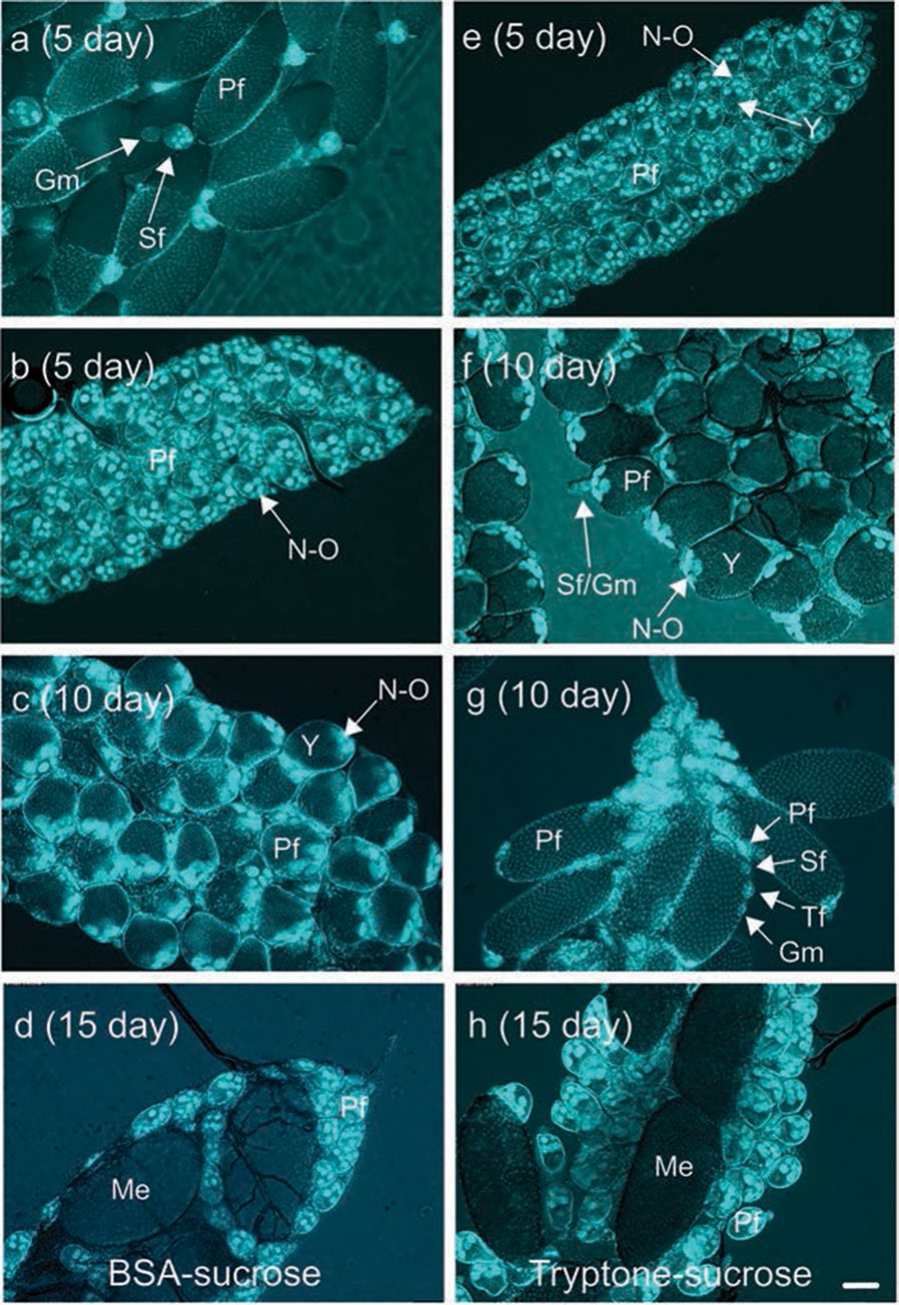
Micrographs of representative ovaries from *Ae. aegypti* females provided ad libitum access to BSA- **a**–**d** or tryptone-sucrose **e**–**f** beginning on day 1 post-emergence. Ovaries were explanted from individual females, fixed and stained with DAPI to visualize nurse cell and oocyte nuclei (N–O) in each follicle. **a** Ovary from a 5-day-old female showing that each ovariole consists of a primary follicle (Pf) fully packaged with yolk, a secondary follicle (Sf) and the germarium (Gm). **b** Ovary from a 5-day-old female that oviposited eggs on day 4, which results in the Sf becoming a Pf containing little yolk but with nurse cell and oocyte nuclei (N–O) readily visible but no tertiary follicle fully developed. **c** Ovary from a 10-day-old female in which most Pfs contain visible nurse cell and oocyte nuclei and intermediate amounts of yolk. **d** Ovary from a 15-day-old female in which some ovarioles contain a mature egg (Me) while others contain a small Pf with little or no yolk. **e** Ovary from a 5-day-old female in which each Pf contains visible nurse cell and oocyte nuclei and small amounts of yolk. **f** Ovary from a 10-day-old female in which nurse cell and oocyte nuclei are visible in most Pfs that contain intermediate amounts of yolk. Some ovarioles also contain newly formed Sfs proximal to the Gm. **g** Ovary from a 10-day-old female in which some ovarioles contain a Pf fully packaged with yolk while other ovarioles contain a Pf, Sf and tertiary follicle (Tf) proximal to the Gm. **h** Ovary from a 15-day-old female in which the ovary contains several Mes plus Pfs with little or no yolk. Scale bar in **h** equals 50 m

The paired ovaries of *Ae. aegypti* are subdivided into 50–60 ovarioles that each contain a primary follicle at emergence [35]. We dissected the ovaries from females fed BSA- and tryptone-sucrose 5–20 days post-emergence followed by fixation and staining with DAPI to visualize the nuclei of the seven nurse cells, the oocyte and enveloping follicle cells. At 5 days, most ovarioles in females fed BSA-sucrose contained a mature primary follicle (≥ 400 µm yolk, chorion present), a secondary follicle and a germarium, whereas the ovarioles in females that had just oviposited predominantly contained early-stage primary follicles with little or no yolk but readily visible nurse cell and oocyte nuclei (Fig. 5a, b). At 10 and 15 days, the ovarioles of some females fed BSA-sucrose contained primary follicles with intermediate but relatively uniform amounts of yolk (~ 200 µm), while the ovarioles of other females contained a small number of mature primary follicles plus a larger number of small primary follicles with little or no yolk (Fig. 5c, d). The ovarioles of most females fed tryptone-sucrose contained primary follicles with little or no yolk at 5 days (Fig. 5e), which was consistent with no females laying eggs at this time. In contrast, 10- and 15-day old females fed tryptone-sucrose exhibited two different states of vitellogenesis. Some individuals contained a predominance of primary follicles with intermediate amounts of yolk plus visible secondary follicles, whereas in other individuals 4–12 ovarioles contained a mature primary follicle, a secondary follicle and a germarium, while other ovarioles contained a small primary follicle with little or no yolk, a secondary follicle and sometimes a tertiary follicle (Fig. 5f, g, h). These results indicated that most females fed BSA-sucrose deposited yolk into oocytes after laying a first clutch but relatively few individuals laid additional eggs, while most females fed tryptone-sucrose contained 50–60 primary follicles per ovary but only some developed into mature eggs that females laid.

### Ad libitum access to protein-sugar meals extends the vitellogenic phase in *Ae. aegypti*

**Fig. 6:**
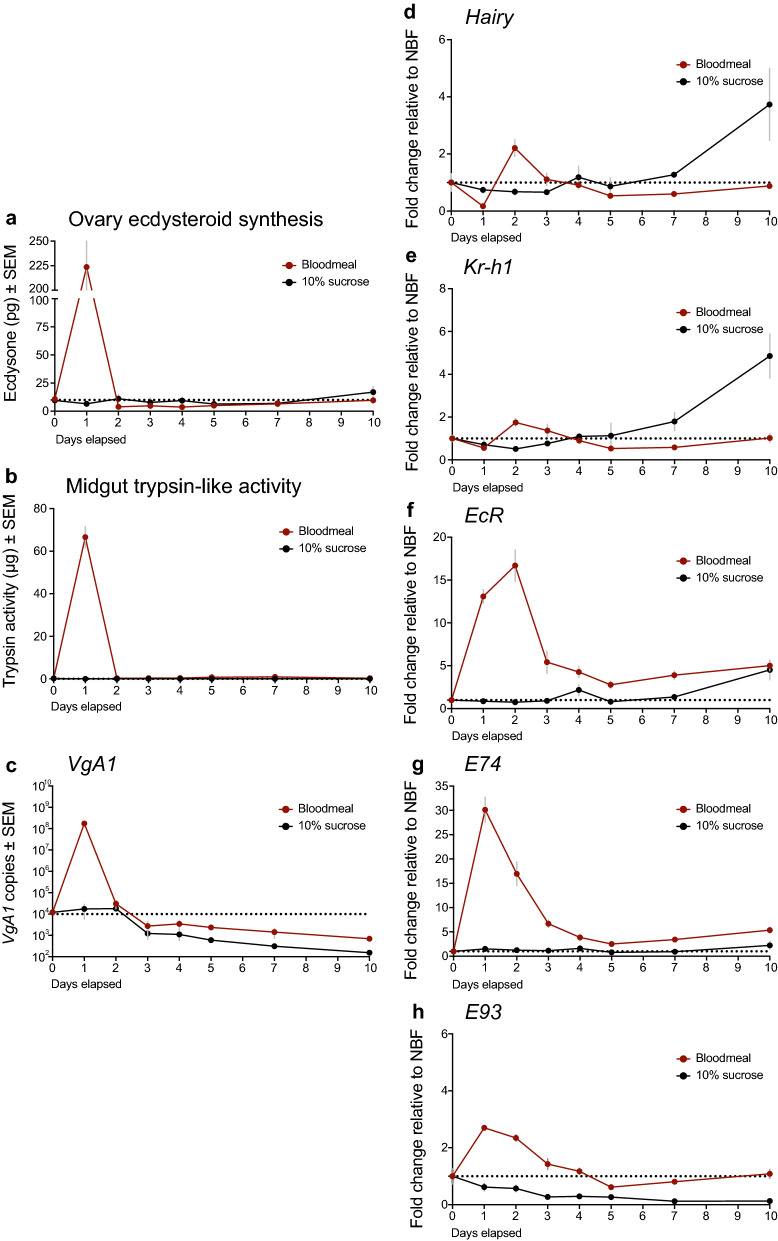
Blood-feeding on day 4 post-emergence activates multiple markers of the vitellogenic phase in *Ae. aegypti* while ad libitum access to 10% sucrose does not. Each marker was measured on the day of blood-feeding and at 24-h intervals thereafter with age-matched sugar fed females measured at the same time: **a** ovary ecdysteroid production measured for 6 ovary pairs per time point; **b** trypsin-like serine protease activity per midgut measured for 5 midguts per time point; **c** *VgA1* copy number measured for three abdomen pairs per time point. Relative expression of the JH responsive genes *Hairy* (**d**) and *Kr-h1* (**e**) and 20E-responsive genes *EcR* (**f**), *E74* (**g**) and *E93* (**h**) normalized to the reference gene *RPL8* and compared to expression levels in 4-day-old sugar-fed control females. Treatment means are shown with error bars (gray) indicating standard error. The dotted horizontal line in each graph indicates *y* = 1 to facilitate visual comparison between control and treatment expression levels

Markers for the previtellogenic phase in *Ae. aegypti* include elevated expression of two JH responsive transcription factors, *Hairy* and *Kr-h1*, which are implicated in regulating JH-mediated functions [59, 60]. Markers for the vitellogenic phase include: (i) a rise in ecdysteroid titer induced by OEH and ILP release from the brain, (ii) upregulated expression of the ecdysone receptor (*EcR*) plus ecdysone-induced proteins *E74* and *E93* that are 20E responsive in the fat body, (iii) upregulated expression of vitellogenin in the fat body and (iv) increased trypsin-like protease activity in the midgut associated with blood meal digestion [32, 38, 39, 46, 51]. Profiling these markers in blood-fed females (positive control) confirmed that *Hairy* and *Kr-h1* expressions were elevated before blood-feeding, declined by 24 h PBM and transiently rose at 48 h PBM, while ecdysteroid titer and midgut trypsin activity rose after a female blood-fed (Fig. 6). The rise in ecdysteroid titer was also mirrored by upregulated expression of *EcR, E74, E93* and *VgA1* in the fat body (Fig. 6). In contrast, when sucrose-fed females (negative control) were examined, *Hairy* and *Kr-h1* expression remained largely unchanged except at very late time points when expression of both rose (days 7–10), while as expected no markers for the vitellogenic phase were upregulated (Fig. 6).

**Fig. 7:**
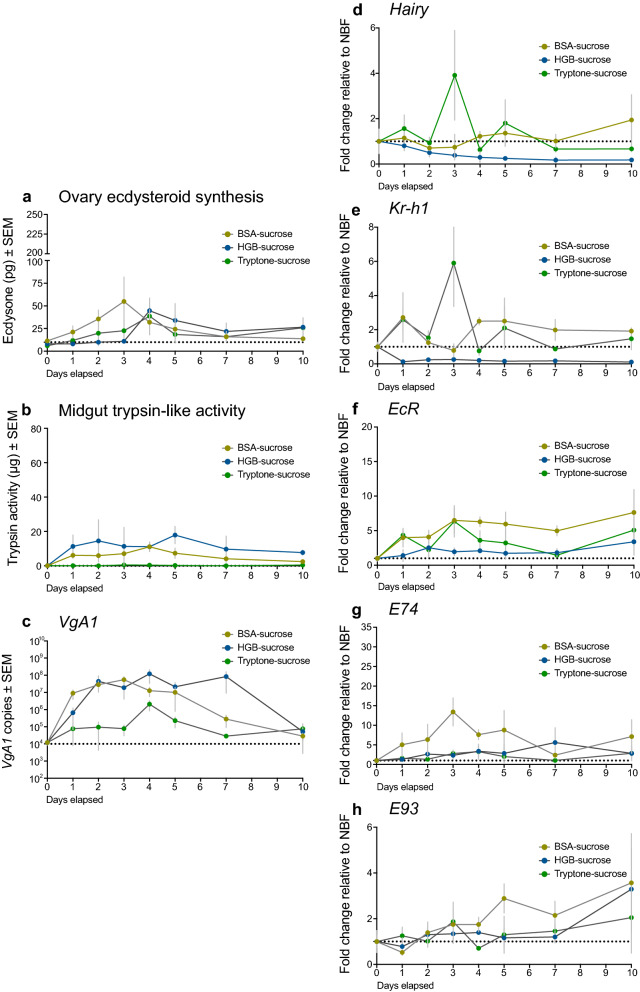
Ad libitum access to BSA-, HGB- or tryptone-sucrose variably activates markers of the of the vitellogenic phase in *Ae. aegypti*. Females were 4 days post-emergence when each feeding treatment began, which is indicated as day 1 on each graph. Each marker was then measured on the first day females were fed and at 24-h intervals thereafter. **a**–**h** Same markers as measured in blood- and sugar-fed control females as defined in Fig. 6

We then examined females provided BSA, HGB or tryptone-sucrose solutions ad libitum. Ecdysteroid titers increased in each treatment but more slowly and with lower maxima than blood-feeding (Fig. 7a). Trypsin-like protease activity increased in the midgut of females fed BSA and HGB-sucrose to levels that were also lower than in blood-fed females while no trypsin-like activity was detected in females fed tryptone-sucrose (Fig. 7b). *VgA1* expression in females mirrored the protracted increase in ecdysteroid titer with copy number rising to a similar maximum in BSA- and HGB-fed females (~ 10^8^) compared to blood-fed females, whereas copy number rose to a lower level (~ 10^5–6^) in females fed tryptone-sucrose (Fig. 6, 7). *Hairy* and *Kr-h1* expression exhibited variable expression patterns with overall declines in HGB-sucrose fed females, overall increases in tryptone-sucrose fed females and intermediate values in BSA-sucrose fed females (Fig. 7d, e). Expression patterns for the 20E responsive genes (*EcR, E74* and *E93*) largely mirrored the protracted increase in 20E titers but were overall lower in females fed HGB- or tryptone-sucrose than BSA-sucrose (Fig. 7f, g, h).

**Fig. 8:**
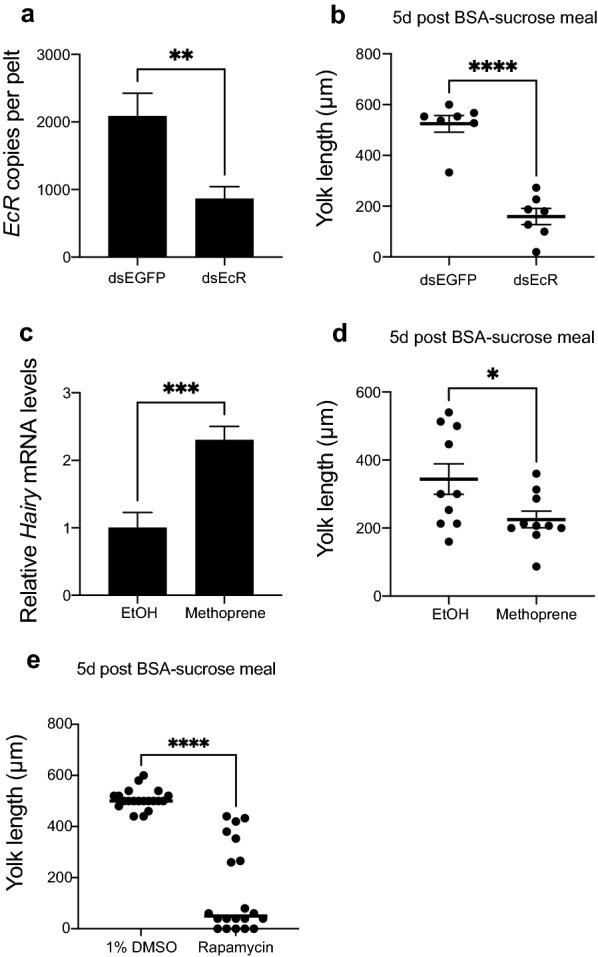
RNAi knockdown of *EcR*, topical application of the JH analog methoprene and feeding of the TOR inhibitor rapamycin disable oocyte maturation when *Ae. aegypti* females are fed BSA-sucrose. **a**, **b** Females injected with dsEcR RNA exhibit a significant reduction in *EcR* transcript abundance and yolk deposition into oocytes compared to females injected with dsEGFP. **c**, **d** Females topically treated with methoprene (250 ng) in ethanol exhibit a significant increase in transcript abundance of the JH-responsive gene *Hairy* but reduced yolk deposition into oocytes compared to females topically treated with ethanol (EtOH) alone. **e** Females that consume BSA-sucrose containing rapamycin dissolved in DMSO deposit significantly lower amounts of yolk into oocytes than females that consume BSA-sucrose containing an equivalent amount of DMSO alone. Yolk length was measured in individual treatment and control females as described in Fig. 1

Given the essential role of 20E in stimulating the vitellogenic phase after blood-feeding, we injected females with dsRNAs that targeted the *EcR* (dsEcR) or *EGFP* (dsEGFP; negative control) 24 h after emerging as adults that only had access to water. Treated females were then provided ad libitum access to BSA-sucrose and water at 48 h followed by assessment of egg formation 5 days later. *EcR* mRNA abundance was significantly reduced by dsEcR, while follicle development as measured by yolk deposition length was also much lower with no mature eggs present in treatment females (Fig. 8a, b). Since *Hairy* and *Kr-h1* expression remained steady state or even slightly increased in females fed BSA-sucrose, we treated newly emerged females with the JH analog methoprene in ethanol, which resulted in increased *Hairy* expression 24 h post-treatment, while yolk deposition into primary follicles was lower at 5 days compared to females topically treated with ethanol alone (Fig. 8c, d). Methoprene-treated females pooled and allowed to oviposit also laid fewer eggs than control females (methoprene treated: 293 eggs/50 females, ethanol only treated: 970 eggs/50 females). Lastly, since egg formation depends on nutrient signaling through the target of rapamycin (TOR) pathway [32, 35, 39], we fed the TOR signaling inhibitor rapamycin [61] to females provided BSA-sucrose. Results indicated that rapamycin also greatly reduced yolk deposition into oocytes (Fig. 8e). Thus, ad libitum consumption of BSA- or tryptone-sucrose stimulated a protracted vitellogenic phase that was disabled by *EcR* knockdown, increased JH signaling via methoprene application and reduced TOR signaling by consumption of rapamycin.

### Ad libitum access to protein-sugar meals reduces host-seeking behavior in *Ae. aegypti*

**Fig. 9:**
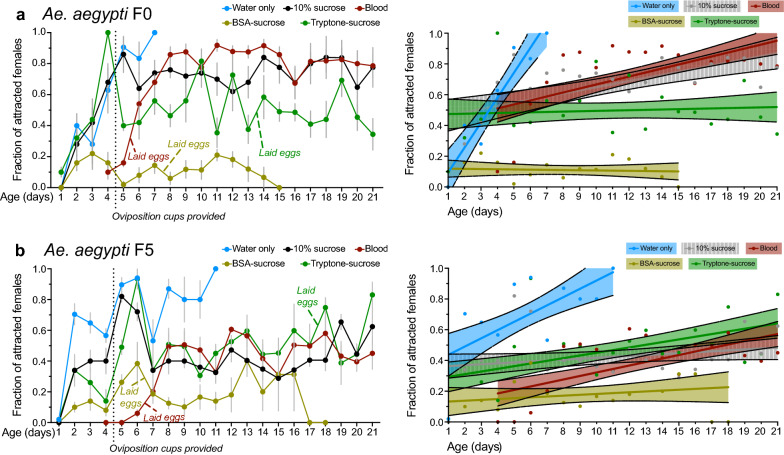
Females provided ad libitum access to BSA- or tryptone-sucrose exhibit a persistent reduction in attraction to a human hand. **a** Newly eclosed *Ae. aegypti* females (F0) were tested for attraction to a human hand daily for 3 weeks when fed water only, 10% sucrose, blood, BSA-sucrose or tryptone-sucrose. **b** *Aedes aegypti* females maintained for five generations (F5) on BSA-sucrose exclusively were similarly tested for attraction for the same treatments as in (**a**). For each treatment, five replicate cages containing ten females each were used for the assay. In the left panel, dots indicate the mean proportion of females that flew to the top of the cage and began probing within 1 min of hand presentation; error bars (gray) indicate standard error. Oviposition substrates were offered when females were 4 days old, and day of egg laying by protein-fed females is indicated. In the right panel, graphs present the same daily means (dots) but overlaid with color-coded slopes indicating linear regression analysis of the mean proportion of attracted females over time, with shaded areas bound by dotted lines indicating upper and lower 95% confidence intervals

Anautogenous mosquitoes are attracted to vertebrate hosts by a variety of long- and short-range cues including carbon dioxide, heat and volatile odorant molecules generated by the host [62]. Mosquito attraction to hosts markedly declines up to 48 h after blood-feeding, coinciding with the vitellogenic phase, while attraction rapidly increases after egg laying [63–66]. Multiple factors are implicated in cessation of host-seeking in blood-fed females including abdominal distention, nutrient acquisition, satiety mediated by neuropeptide Y signaling and upregulated expression of *vitellogenin* [56, 67–69]. Since *Ae. aegypti* females exhibited a protracted vitellogenic phase when provided ad libitum access to BSA- or tryptone-sucrose, we asked if newly emerged females (F0) from our general culture exhibited differences in their attraction to humans when maintained in this way by measuring probing behavior over a 3-week period. Results were then compared to other cages in which females were blood-fed, provided ad libitum access to 10% sucrose solution and water, or provided ad libitum access to water alone. A repeated measures analysis indicated that treatments were significantly different (F = 20.886, df = 4, *p* < 0.0001) (Fig. 9a). As expected, host attraction rapidly increased after emergence for females provided with water alone but these individuals died earlier than in the other treatments (Fig. 9a). Females provided 10% sucrose also exhibited strong host attraction that remained elevated over the 3 weeks we conducted assays (Fig. 9a). Females that blood-fed when 4 days old exhibited little probing behavior up to 48 h followed by a rapid increase in the proportion of females that probed persistently for the remainder of the assay (Fig. 9a). In contrast, tryptone-sucrose-fed females exhibited moderate host attraction with ~ 50% of females probing while BSA-sucrose -fed females exhibited low host attraction with only ~ 20% of females probing at most sample times (Fig. 9a).

We next asked if intergenerational maintenance on protein-sucrose meals could induce adaptive behavioral changes in *Ae. aegypti* by assessing whether host-seeking behavior differed in females after five generations (F5) of being maintained on exclusively BSA- or tryptone-sucrose plus water. F5 female attraction to a host differed significantly by treatment over the course of the study (F = 6.409, df = 4, *p* < 0.0001) but trends of attraction for each treatment were overall similar to F0 females with BSA-sucrose-fed females exhibiting exceptionally low attraction (Fig. 9b). Blood-fed F5 females displayed reduced attraction and probing compared to blood-fed F0 females; however, we detected no statistical difference in the proportion of 4-day-old F0 and ensuing F6 generation females that actually fed to repletion when offered an anesthetized rat or a heated membrane feeder containing rabbit blood plus ATP (Table 3). F0 and F6 females that fed to repletion took the same amount of time to oviposit when fed rabbit blood, while F5 females took roughly 1 day longer to lay eggs after blood-feeding on a rat than F0 females. The total number of eggs laid by F0 and F5 females after blood-feeding also did not differ. We thus concluded that females given ad libitum access to BSA- or tryptone-sucrose exhibit lower host attraction than females fed sucrose alone. In contrast, feeding females BSA-sucrose for five generations did not reduce their avidity to blood-feed on a mammal or membrane feeder.

**Table 3:** Response to blood meal by *Ae. aegypti* F0 versus tryptone-sucrose F6

	Live rat		Defibrinated rabbit blood		
	F0	F6	F0	F6	
Proportion of replete females	83/99 (84%)	71/96 (74%)^ns^	81/99 (90%)	83/100 (83%)^ns^	
Time taken to oviposit (days)	3.8 ± 0.9	5.1 ± 1.8**	3.3 ± 0.9	3.4 ± 1.0^ns^	
Total eggs per female	91.3 ± 63.6	93.5 ± 60.8^ns^	119.6 ± 49.6	99.2 ± 53.5^ns^	

Pairwise statistical comparisons of F0 and F6 females are indicated to the right for both treatments. Fisher’s exact test was used to compare proportion of replete females (row 1) and Student’s *t*-test used to compare time to oviposit and egg clutch size (rows 2 and 3). *ns,* not significant; *p < 0.05, **p < 0.01, ***P < 0.001, ****P < 0.0001

## Discussion

Mosquitoes reside in the order Diptera (flies) where egg formation in species like *Drosophila melanogaster* and *Musca domestica* is also affected by diet and hormones [70–72]. However, anautogenous mosquitoes differ from most other dipterans because blood-feeding activates vitellogenesis, which results in females producing eggs in discrete gonadotrophic cycles that are tightly coupled to locating hosts [32–35]. Mosquitoes also readily consume sugar meals that females distinguish from blood through neurons on the mouthparts used for feeding [73]. Sugar consumption is thought to primarily extend adult longevity and provide energy for flight [6]. However, some anautogenous species may initiate egg formation in the field without blood-feeding given: (i) plant nectars often contain amino acids or proteins [3–6] and (ii) results reported here and elsewhere [26–31] showing that some species in culture produce eggs when fed sugar solutions containing different sources of amino acids. Our goal in conducting this study was to explore how sucrose solutions containing different sources of amino acids affect the number of eggs females produce and the processes that regulate oogenesis. We focused on *Ae. aegypti* because egg formation has been most studied in this species but also conducted assays with four other species to assess whether responses were similar.

We initially fed starved UGAL *Ae. aegypti* a single protein-sugar meal to repletion because prior studies using artificial blood meals or protein-sugar solutions have fed females in this way [19, 21, 25, 29–31]. However, this approach mimics blood-feeding more than nectar feeding in the field or sugar feeding in laboratory, which is why we also conducted assays where females had ad libitum access to protein-, peptide- or amino acid-sugar solutions [6]. Our single meal assays indicate UGAL *Ae. aegypti* produce similar numbers of eggs after feeding to repletion on 20% BSA-10% sucrose or rabbit blood from a membrane feeder. This outcome was expected given results showing that *Ae. aegypti* produce similar numbers of eggs after consuming a blood meal, 10–20% BSA plus ATP or solutions containing multiple proteins plus ATP from a membrane feeder [16, 19, 21, 22]. *Aedes albopictus* females also produce similar numbers of eggs after feeding on a host or consuming a meal of 10% BSA-sucrose [31]. Our results indicate *Ae. aegypti* produce no mature eggs after consuming a single tryptone- or amino acid-sucrose meal but ad libitum access to the former results in mature eggs after 8 days while the latter results in some yolk deposition after 10 days. We thus conclude UGAL *Ae. aegypti* cannot produce eggs by consuming sugar solutions containing amino acids but nectars containing amino acids could accelerate egg maturation or average clutch sizes by stimulating some yolk deposition into primary follicles before a blood meal. The inability to produce mature eggs when fed an amino acid-sucrose solution and slower maturation of eggs when fed tryptone-sucrose are also consistent with results suggesting amino acids and peptides may be excreted too rapidly to be efficiently absorbed by the midgut [16, 74]. That blood and BSA strongly upregulate digestive enzymes but tryptone does not could also contribute to lower levels of nutrient uptake. In mammals, proteins are digested into peptides and amino acids that are absorbed by several types of oligopeptide and amino acid transporters [75, 76]. Homologs to some of these transporter families have been identified in insects including *Drosophila* and mosquitoes [76, 77] but most remain functionally uncharacterized. On the other hand, our results suggest nectars containing protein could stimulate egg formation. Genetic factors also affect autogeny [9–12]. Thus, responses to peptide- or amino acid-sugar solutions could also differ among strains of *Ae. aegypti* given results showing that a field-collected population of *Ae. aegypti* produced some mature eggs autogenously when nutrients consumed by larvae or adults were increased [15].

Our comparative studies indicate *Ae. albopictus* females produce similar numbers of mature eggs as *Ae. aegypti* when provided ad libitum access to BSA-sucrose, whereas lower proportions of *An. gambiae, An. stephensi* and *C. quinquefasciatus* females do so. As earlier noted, *Ae. albopictus* females were previously shown to lay eggs after consuming a single BSA-sucrose meal, but smaller proportions of *An. darlingi* females laid eggs when fed similarly [31, 32]. These findings together with our results thus suggest BSA more strongly promotes egg formation in *Aedes* than *Anopheles* or *Culex* spp. *Culex quinquefasciatus* feeds on mammals including humans but several studies indicate a preference for blood-feeding on birds [78, 79] while other results suggest *C. quinquefasciatus* and other *Culex* spp. reproductively benefit more from avian than mammalian blood [16, 80–85]. Other proteins besides BSA or other unknown factors in avian blood may thus be involved in activating the vitellogenic phase in *C. quinquefasciatus*. We previously identified no differences in egg formation by *An. gambiae* when fed to repletion on blood from humans, other mammals or birds despite being anthropophilic [16]. *Anopheles gambiae* and several other *Anopheles* spp. are also known to sometimes consume more than one blood meal per gonadotrophic cycle [86–88] but our results indicate that ad libitum access to BSA-sucrose still results in many females not maturing eggs. We thus speculate that other factors such as the amount of 20E obtained from males during mating [89] or lower teneral nutrient reserves potentially contribute to why *An. gambiae* and *An. stephensi* mature fewer eggs than *Ae. aegypti* and *Ae. albopictus*.

Our intergenerational rearing studies indicate UGAL *Ae. aegypti* can be maintained using BSA-, HGB- and tryptone-sugar meals instead of blood-feeding. BSA-sucrose was previously shown to also support intergenerational rearing of *Ae. albopictus*, which overall suggests protein-sugar meals may be attractive for rearing several *Aedes* spp. because they eliminate the need for temperature-regulated artificial blood meals, membrane feeders or live hosts [31]. The intergenerational increase in hatch rates when UGAL *Ae. aegypti* females were fed BSA- or tryptone-sucrose further suggests genetic variation exists in egg development and/or the viability of first instars after hatching that can be selected for despite long-term rearing of this strain by blood-feeding. Our decision to add supplemental iron to BSA- and tryptone-sucrose may also promote hatching as previously reported for some artificial blood meals [17, 22]. In contrast, our results from *An. gambiae, An. stephensi* and *C. quinquefasciatus* suggest conditions used in this study will require further optimization for intergenerational rearing, which is also suggested by results for *An. darlingi* [30].

An additional takeaway from our study is that the vitellogenic phase is greatly prolonged when mosquitoes continually consume peptides or proteins in sugar solutions. Prior studies strongly support essential roles for JH, ILPs, OEH and 20E in regulating gonadoptrophic cycles after *Ae. aegypti* females blood-feed [32–36, 39]. Multiple JH and 20E responsive genes have also been identified that affect vitellogenin expression early in the vitellogenic phase or inhibit expression in the late phase [32, 33, 46, 59, 60]. Consistent with this literature, our control assays show that ecdysteroid production by ovaries contributes to the rapid rise in hemolymph 20E titer after females blood-feed, which is followed by a rapid rise in *VgA1* expression followed a decline in *VgA1* expression before mature eggs are laid. Ad libitum access to BSA-, HGB- or tryptone-sucrose in contrast extends the vitellogenic phase, as evidenced by ecdysteroid production remaining elevated, several 20E responsive genes being persistently upregulated and *VgA1* being persistently expressed. Our results also reveal an interesting phenotype associated with long-term ingestion of protein-sugar meals: precocious development and yolk deposition into secondary or tertiary follicles concurrent with the maturation of primary follicles. This phenomenon was also noted in *Ae. aegypti* and *An. stephensi* injected with large amounts of ecdysone or 20E [90, 91], lending further support that peptide- or protein-sugar-fed females undergo continual ecdysteroidogenesis and protracted vitellogenesis.

Contrary to expectation though, our results indicate most *Ae. aegypti* females provided BSA-sucrose ad libitum lay only one clutch or two smaller clutches over consecutive days in a manner similar to females that consume a single blood meal. In addition, females provided HGB-sucrose or tryptone-sucrose ad libitum lay fewer eggs in total that are oviposited later as a single clutch or several smaller clutches. The delays in egg formation associated with tryptone-sugar feeding correlate with ecdysteroid production and titers rising more slowly and 20E-responsive genes plus *VgA1* being expressed at lower levels than when females are fed BSA-sugar. Unlike females that were fed BSA-sugar ad libitum, females fed tryptone-sugar ad libitum also exhibited an increase in expression of JH responsive genes at 3 days. Thus, in addition to tryptone potentially being excreted too rapidly to be efficiently absorbed, increased JH titers in the early phases after females start consuming tryptone-sugar could also potentially contribute to slower maturation of eggs than occurs when females are fed BSA-sugar. We are less clear about our HGB-sugar feeding data that reveal expression patterns more similar to tryptone-sugar feeding but total egg numbers that are more similar to females consuming BSA-sugar solutions.

Our time course studies comparing follicle development further indicate ad libitum access to BSA-sugar leads to maturing a large first of clutch eggs but thereafter fails to produce a normal second clutch, while females provided tryptone-sugar produce fewer primary follicles that fully mature. Thus, while UGAL *Ae. aegypti* was readily maintained on BSA-sucrose over multiple generations with increasing hatch rates, our results also suggest constraints exist that prevent females from producing eggs continuously when consuming protein daily. However, repressed host-seeking behavior when fed BSA-, HGB or tryptone-sucrose ad libitum is consistent with results that implicate abdominal extension and activation of the vitellogenic phase with reduced attraction to human or other vertebrate hosts [56, 67, 68].

Lastly, our results have potential implications for reproduction and dispersal of feral mosquito populations. Laboratory studies of reproductive physiology focus on females consuming a single large blood meal per reproductive cycle, but some vector species in the field ingest multiple small blood meals used in part to fuel flight and replenish metabolic reserves [92]. Similar to our findings, more frequent blood-feeding also increases asynchronous egg formation including repeated oviposition of small egg clutches that can drive mosquito dispersal similar to skip oviposition [93]. More frequent blood consumption also increases chances of pathogen acquisition, midgut escape and transmission [92, 94, 95]. Thus, studies of feral mosquitoes together with findings reported here overall suggest greater plasticity in egg formation than is suggested by studies where females feed to repletion. Our results further suggest environmental conditions and diet may contribute more to facultative autogeny and vectorial capacity in some species than is generally recognized.

## Conclusions

UGAL *Ae. aegypti* females produced similar numbers of eggs when fed a BSA-sucrose solution or a blood meal. In contrast, four other anautogenous mosquitoes produced variable numbers of eggs which indicated egg formation in response to BSA-sucrose feeding was not generalizable. Focusing on UGAL *Ae. aegypti*, functional assays indicated that females also produced mature eggs when provided ad libitum access to HBG or tryptone in sucrose but only deposited small amounts of yolk when provided essential amino acids in sucrose. UGAL *Ae. aegypti* could also be maintained without blood-feeding by provisioning females with BSA-, HGB- or tryptone-sucrose but the average number of eggs individual females produce when fed these solutions varied. Ad libitum access to BSA-, HGB- or tryptone-sucrose stimulated a protracted vitellogenic phase but females notably did not produce eggs continuously. Thus, constraints associated with long-term blood-feeding likely prevent females from being able to fully shift from maturing eggs in discrete gonadotrophic cycles.

## Supplementary Information

**Additional file 1: Table S1. **Primers used in this study.

## Data Availability

All essential data for this study are presented in the main text of the manuscript. Data sets are freely available from the corresponding author upon request.

## Abbreviations

20E: 20-hydroxyecdysone
ATP: Adenosine triphosphate
BSA: Bovine serum albumin
ECD: Ecdysone
EIA: Enzyme-linked immunosorbent assay
HGB: Hemoglobin
ILP: Insulin-like peptide
IR: Insulin receptor
JH: Juvenile hormone
OEH: Ovary ecdysteroidogenic hormone
OEHR: OEH receptor
PBM: Post blood meal
PBS: Phosphate-buffered saline
SD: Standard deviation
SP: Serine protease
TOR: Target of rapamycin
UGAL: University of Georgia strain
Vg: Vitellogenin
