# Supplementary material for: Ad libitum consumption of protein- or peptide-sucrose solutions stimulates egg formation by prolonging the vitellogenic phase of oogenesis in anautogenous mosquitoes
Source: Parasit Vectors. 2022 Apr 12;15:127. doi: 10.1186/s13071-022-05252-4 (PMC9004051; doi:10.1186/s13071-022-05252-4)
Supplement: Supplementary file 1 — Additional file 1: Table S1. Primers used in this study. [file 13071_2022_5252_MOESM1_ESM.docx]

| Target | Direction | Sequence | Reference |
| --- | --- | --- | --- |
| *RPL8* | Forward | 5’ – AAG GGA GAG CCA AAA TTG C – 3’ | Dzaki *et al.* 2017 |
|  | Reverse | 5’ – CAG TAC ACA AAC TGT CCG GTG T – 3’ |  |
| *Hairy* | Forward | 5’ – ACT GTT CGG TGA AGA TCG TG – 3’ | Wang *et al.* 2021 |
|  | Reverse | 5’ – CTC CTC GCA ATT TCA GTC TC – 3’ |  |
| *Kr-h1* | Forward | 5’ – GAA CGA CCA TTC CCA GTC G – 3’ | Wang *et al.* 2021 |
|  | Reverse | 5’ – TGC TGG AAC CTA TGC TGT TG – 3’ |  |
| *EcR* | Forward | 5’ – AAG CGA GGT TAT GAT GTT GCG – 3’ | Wang *et al.* 2017 |
|  | Reverse | 5’ – CAG CAG GTC CTC TAT CGT GTC C– 3’ |  |
| *E74* | Forward | 5’ – GCG AGT ACT GCC CTC GGT TC – 3’ | Wang *et al.* 2021 |
|  | Reverse | 5’ – CAC CGG TCG CTT CTC GTA CC – 3’ |  |
| *E93* | Forward | 5’ – ACT GGA GCG CGT TGC TGA AGA – 3’ | Wang *et al.* 2021 |
|  | Reverse | 5’ – AGG TTC AAC GGT TGG CGC GT – 3’ |  |
| *VgA1* | Forward | 5’ – AGG CGT TCT GAT ATC CAT ATT CG – 3’ | None |
|  | Reverse | 5’ – ACT ACT TCT TCT CGC TTT GGC – 3’ |  |
| *dsEcR* | Forward | t7 – ACT ACA ATG CCC TCA CCT GC | None |
|  | Reverse | t7 – GGT AAA TGC TGG CAG TCC CT |  |

**Table S1. Primers used in this study**
